# The paradox of cellular senescence in prostate cancer: from tumor suppression to tumor promotion

**DOI:** 10.3389/fonc.2026.1743564

**Published:** 2026-02-17

**Authors:** Yitao Xing, Guozheng Qin, Xueying Lin, Siyu Chen, Yan Su, Tiandong Lin, Guifei Chen

**Affiliations:** 1 The First School of Clinical Medicine, Yunnan University of Chinese Medicine, Kunming, Yunnan, China; 2 China Joint Graduate School of Traditional Chinese Medicine, Suzhou, China; 3 Hainan Hospital, Guangdong Provincial Hospital of Chinese Medicine, Haikou, China; 4 The First Affiliated Hospital of Yunnan University of Chinese Medicine, Yunnan Provincial Hospital of Chinese Medicine, Kunming, Yunnan, China; 5 College of Traditional Chinese Medicine, Hainan Medical University, Haikou, China; 6 The Second Affiliated Hospital of Hainan Medical University, Haikou, China

**Keywords:** cellular senescence, proinflammatory cytokines, prostate cancer, senescence-associated secretory phenotype (SASP), tumor microenvironment

## Abstract

Cellular senescence is a fundamental biological program that enforces a stable cell-cycle arrest in response to diverse stresses, acting as a crucial barrier against malignant transformation. In the context of prostate cancer, however, this protective mechanism paradoxically exhibits tumor-promoting potential under certain microenvironmental and therapeutic conditions. This review examines the various aspects of senescence in prostate cancer development, ranging from DNA damage– and oncogene-induced senescence (p53/p21, PTEN/p16, and RAS/MYC) to treatment-induced senescence following androgen deprivation, radiotherapy, and chemotherapy. While senescence temporarily halts tumor formation via cell-intrinsic checkpoints and immune surveillance, overly persistent senescent cells trigger the development of a senescence-associated secretory phenotype (SASP) in which they secrete proinflammatory cytokines, chemokines, and proteases that modify the tumor microenvironment in ways that encourage inflammation, assist in the hyper-epithelial plasticity, and therapeutic resistance of the tumor. This review highlights new insights into the epigenetic control and metabolic rewiring that determine the shift between tumor-suppressing senescence and tumor-promoting senescence. This review consolidates the paradox of ‘double-edged sword’ senescence, delineating that its impact is contextually dependent on the cell, immune environment, and the duration of senescence. Finally, we discuss the predicted therapeutic approaches based on precision oncology to target and alter senescence using senolytics to eradicate aberrant senescent cells and senomorphics to target and adjust SASP. Addressing and regulating the plasticity of senescence is a significant and critical opportunity for improving the prognosis of prostate cancer, as well as guiding next-generation senescence-informed therapeutic approaches.

## 1 Introduction

Cellular senescence represents a state of stable proliferative arrest triggered by a wide range of cellular insults, including telomere shortening, oncogene activation, and genotoxic or oxidative stress. Unlike quiescence, senescence is irreversible and characterized by profound chromatin remodeling, metabolic reprogramming, and secretion of bioactive molecules that reshape the tissue microenvironment (1). These molecular and phenotypic changes are orchestrated through key signaling pathways such as the p53/p21^CIP1 and p16^INK4a/Rb axes, which serve as central effectors maintaining the senescent phenotype. While these pathways prevent the replication of damaged cells, the persistence of senescent cells within tissues can paradoxically contribute to pathophysiological states associated with chronic inflammation and tumor progression (2).

**Figure 1:**
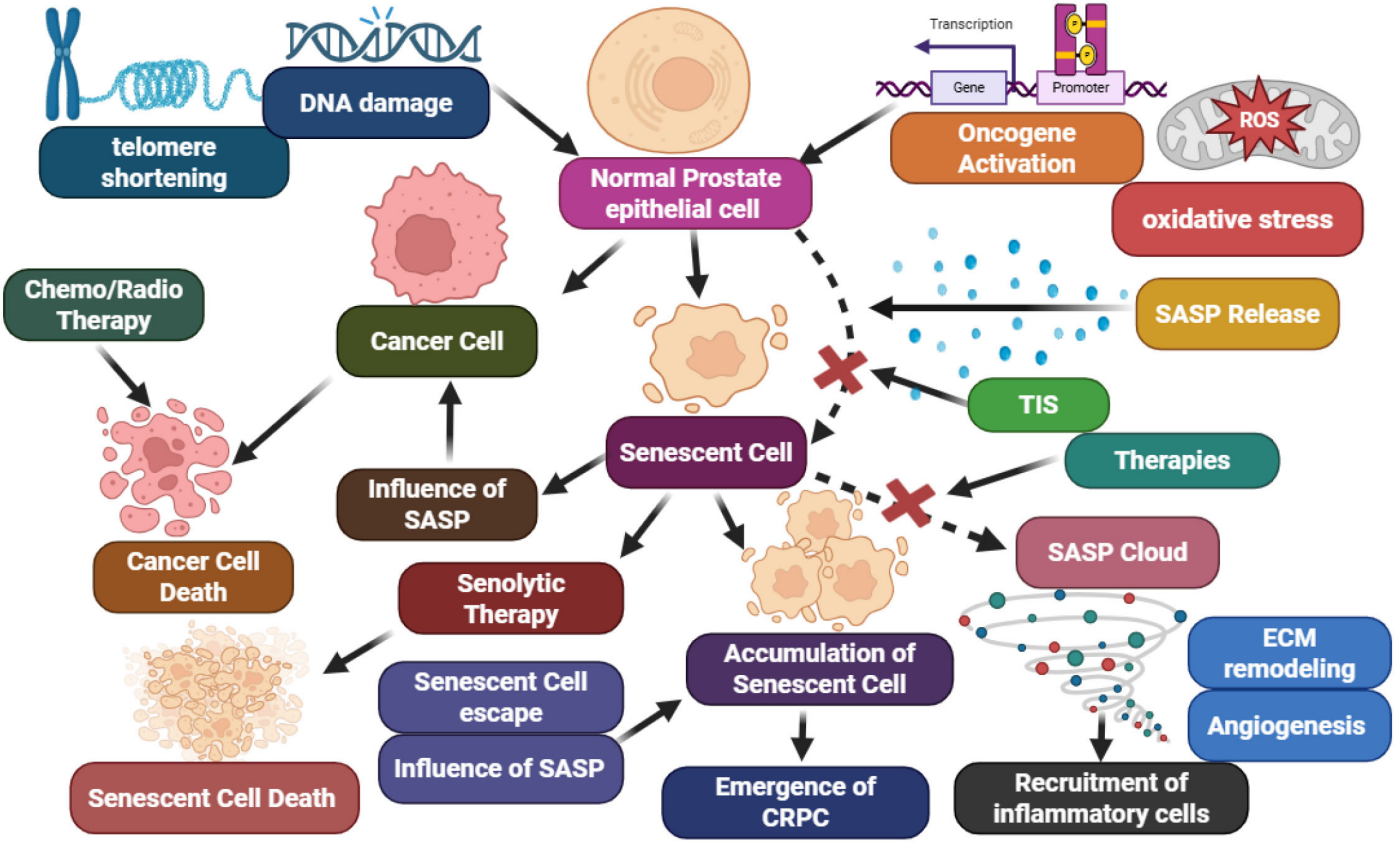
Dual role of cellular senescence in prostate cancer: early senescence halts tumor growth, while persistent or TIS drives SASP-mediated inflammation, angiogenesis, and progression to castration-resistant disease.

Senescence serves an ambiguous and remarkable role in the context of advanced prostate cancer (PCa). During the early stage of tumorigenesis, oncogene- or stress-induced senescence continues to take place as a safeguard against malignant transformation. For example, pre-neoplastic lesions, such as prostatic intraepithelial neoplasia (PIN), display cellular senescence markers, suggesting the phenomenon serves as a biological checkpoint to halt neoplastic progression (3). However, in more advanced disease stages, senescent cells can accumulate within both epithelial and stromal compartments, altering the tumor microenvironment through their secretory activity (4). A key feature of senescent cells is the senescence-associated secretory phenotype (SASP), a complex secretome composed of proinflammatory cytokines (e.g., IL-6, IL-8), chemokines, proteases, and growth factors. While SASP initially promotes immune surveillance and clearance of senescent cells, its persistence can remodel the extracellular matrix, promote angiogenesis, and enhance the survival and invasiveness of adjacent malignant cells. This duality underlies the “senescence paradox” in cancer biology (5). Adding further complexity, emerging evidence suggests that therapy-induced senescence (TIS), a response triggered by radiation, chemotherapy, or androgen deprivation therapy, may temporarily halt tumor growth but also contributes to tumor recurrence through residual SASP-driven inflammation and stemness reprogramming (6, 7). This adaptive evolution highlights the intricate interplay between senescence signaling and therapeutic outcomes (**Figure 1**).

This review explores the molecular mechanisms, context-dependent effects, and therapeutic implications of senescence in prostate cancer. We examine how DNA damage responses, oncogene signaling (e.g., MYC, RAS, PTEN), and therapeutic stress interact with senescence pathways. Furthermore, we evaluate emerging strategies to therapeutically modulate senescence, including senolytic agents that selectively eliminate senescent cells and senomorphics that suppress SASP without affecting cell-cycle arrest. We conclude with a discussion on the potential of senescence-informed precision oncology, highlighting challenges in biomarker development, therapy resistance, and future research directions.

## 2 Mechanisms of cellular senescence in prostate cells

**Figure 2:**
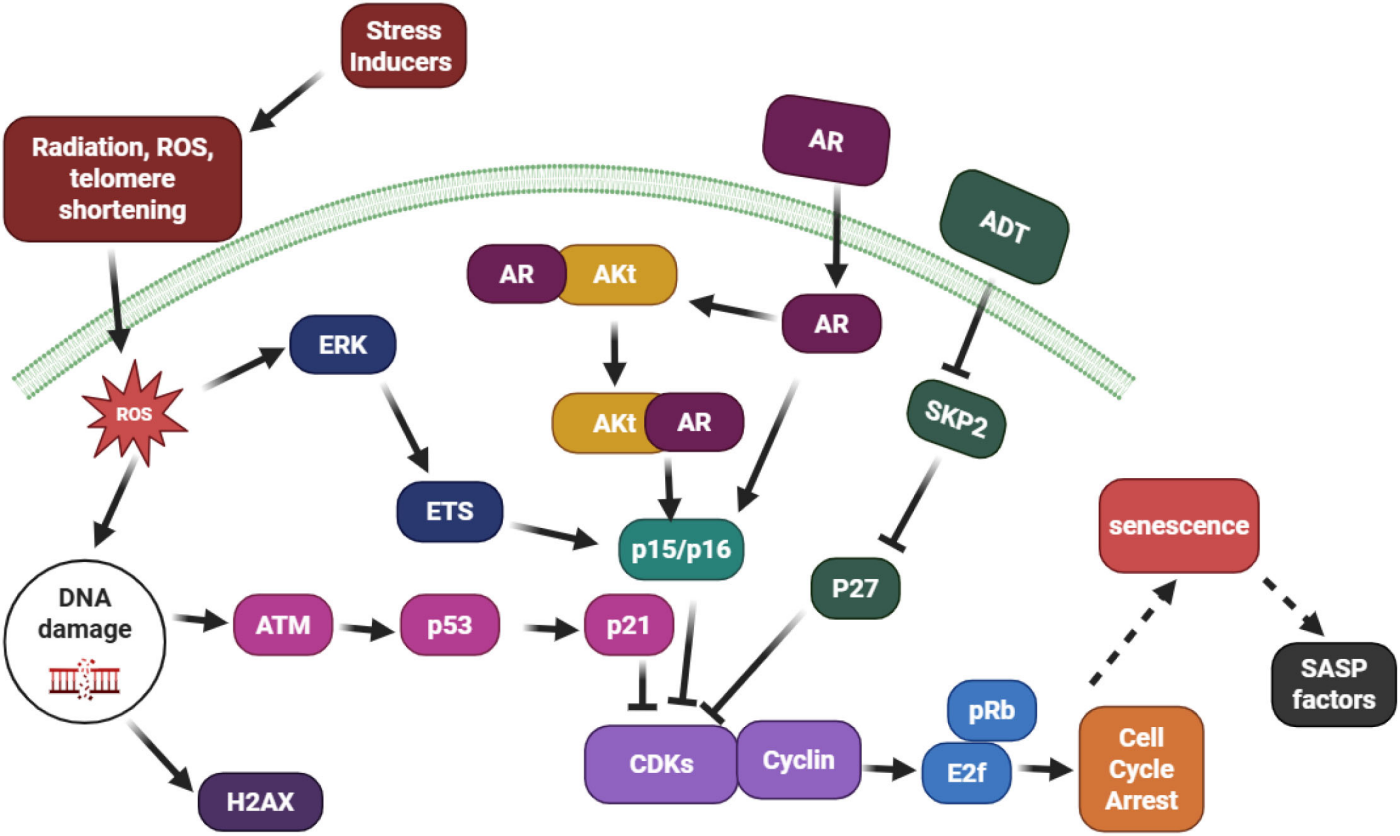
Mechanistic overview of cellular senescence in prostate cancer. Diverse stimuli DNA damage, oncogenic activation, therapeutic stress, and epigenetic remodeling, converge on the p53/p21, p16/Rb, and PTEN/p27 axes to enforce cell-cycle arrest. While initially tumor-suppressive, persistent senescent cells secrete SASP factors that drive inflammation, therapy resistance, and tumor progression.

Cellular senescence in prostate cancer represents a multifactorial process governed by diverse genetic, epigenetic, and therapy-induced perturbations that converge on irreversible cell-cycle arrest (**Figure 2**). The senescent phenotype in prostate cells emerges primarily from DNA damage–mediated stress, oncogene activation, cytotoxic therapies, and chromatin remodeling events. These mechanisms are highly context-dependent, initially enforcing tumor suppression, yet paradoxically contributing to tumor progression once clearance of senescent cells fails (8).

### 2.1 DNA damage–induced senescence (p53/p21 pathway)

The p53-p21 signaling pathway is the canonical backbone of prostate epithelial cell stress-induced senescence. Genotoxic stress, whether caused by oxidative stress, telomere shortening, or radiation, triggers the DNA damage response (DDR), ATM and ATR phosphorylation, and p53 stabilization. Activated p53 transactivates p21^CIP1/WAF1, which inhibits the cyclin E–CDK2 complexes and keeps Rb in its hypophosphorylated, growth-suppressive, and active form. This, in turn, causes the E2F-dependent transcription to be silenced and leaves the cell in a G1-phase of the cell cycle permanently (9, 10).

In early prostatic intraepithelial neoplasia (PIN), this mechanism is the critical malignancy checkpoint. However, in later stages of the disease, TP53 loss or mutation and CDKN1A silencing diminish this checkpoint, which drives proliferation and unstable genomes. Additionally, DDR in fully senescent prostate cells is sublethal and activates SASP via the NF-κB and cGAS–STING pathways, which fosters an inflammatory niche. This inflammatory signaling is initiated when cytoplasmic chromatin fragments activate the cGAS–STING axis, which in turn triggers NF-κB translocation and promotes transcription of SASP genes. This cascade sustains low-grade inflammation and contributes to tumor progression, particularly in the context of therapy-induced senescence, where residual cells persist (11).

### 2.2 Oncogene-induced senescence (RAS, MYC, PTEN loss)

Prostate epithelial cells exhibit a well-ordered response to stress induced by oncogenic signaling. RAS or MYC hyperactivation, or loss of PTEN, triggers an anti-proliferative senescent program that includes the activation of ARF–p53–p21 and p16–Rb. During the early stages of tumorigenesis, this response serves as an intrinsic barrier, arresting cells that have acquired an excessive proliferative signal (12). Losing PTEN, a lipid-phosphatase and negative regulator of the PI3K–AKT–mTOR pathway, is of special importance to prostate cancer. PTEN-deficient cells losing PTEN will initially senesce due to the PTEN loss senescence program (PICS), which is attributed to the upregulation of p27^Kip1 and the downregulation of cyclin D1, thereby G1–S transition is restricted. However, persistent AKT–mTOR signaling following PTEN loss promotes metabolic reprogramming and oxidative stress, which ultimately enables the cell to bypass senescence. This shift fosters clonal expansion, resistance to therapy, and supports the emergence of aggressive, stem-like prostate cancer cells (13). Likewise, aberrant expression of MYC will induce replicative stress, which will recessively activate p53 and p16 and the p16 checkpoint; with time, these pathways will adapt and convert the senescence barrier to a pro-tumorigenic SASP state (7).

### 2.3 Therapy-induced senescence in prostate cancer

TIS occurs as a result of treatment strategies like chemotherapy, radiotherapy, and androgen-targeted therapy. One of the many consequences of TIS is the treatment of prostate cancer. Under the circumstances of chemotherapy, the goal of therapy is not to kill the cancer. Rather, the objective is to reprogram the tumor to a state of perpetual arrested proliferation. This state is defined by an activated DNA damage response, a reprogrammed chromatin, and a senescence-associated secretory inflammatory response (8, 14).

In geriatric oncology, the senescence mechanism of action of chemotherapeutic agents, especially docetaxel, doxorubicin, and mitoxantrone, is predicated on DNA damage and oxidative stress. The outcome of their action is the upregulation of the CDK inhibitors, p21^WAF1/CIP1 and p16^INK4A, which in turn culminates in the cyclin-dependent kinase (CDK) inhibition and hypophosphorylation of the retinoblastoma (15) tumor suppressor, which also plays a role in the LNCaP, DU145, and PC3 cells (16). Senescence and reactivation of transcription of tumor suppressor genes, which result in chromatin expansion and accessibility, are also consequences of cancer therapies that employ DNA methyltransferase inhibitors like 5-azacytidine. In the broader context of oncology and PCa, chemotherapy-induced senescence may work as a tumor-suppressive mechanism, even as the inflammation-driven SASP fuels therapy resistance (17).

Radiation therapy (RT) is a very effective treatment modality for localized and recurrent PCa. However, the antitumor effect of RT is not limited to cytotoxicity (17). Prostate epithelial and cancer cells exposed to gamma or X-irradiation (2-10Gy) experience double-strand breaks, ATM-p53-p21 axis triggering, and G1/S phase arrest, leading to senescence and not apoptosis (18). Studies show that p53-competent cell lines such as LNCaP and 22Rv1 experience significantly higher senescence rates, while p53-deficient DU145 cells radiation radiation-induced senescence almost 0, which emphasizes the role of the p53/p21 axis in radiation-induced senescence (18). However, higher doses of irradiation can also activate p53-independent mechanisms, particularly through p16^INK4 and p107/p130 pocket proteins. It has also been shown that temporarily stabilizing p53 in prostate cancer cells using pharmacologic agents such as Nutlin-3 radiation-augments p21 senescence, which renders cells more sensitive to p21 levels. This underscores the point that senescence triggered through radiotherapy is not only p53-dependent. There are undoubtedly other mechanisms involved in ensuring p53-dependent senescence is arrested (8).

Androgen antagonist therapy of bicalutamide, enzalutamide, and darolutamide, as well as ADT, triggers cellular senescence by modulating cell-cycle inhibitors. In the LNCaP and LAPC-4 ADT performed models, C/EBPβ activation and subsequent oxidative stress ADT triggers result in upregulation of p27^Kip1 and p16^INK4A. They promote senescence through the pRb hypophosphorylated state and cyclin D1 suppression. Loss of PTEN appears to be one of the principal factors in the senescence bypass described just above. Loss of PTEN function decreases p27 activity, thereby promoting senescence escape, while restoration of PTEN increases sensitivity to AR-targeted therapy (19). Evidence summarized in **Table 1** from experimental and clinical models demonstrates that diverse therapeutics, including DNA-damaging agents, radiation, and hormonal inhibitors, act on these tumor-suppressing signaling axes to establish a senescent phenotype. Activating overlapping pathways of p53/p21, p16/pRb, and PTEN/p27 signaling drives the therapy-induced cell-cycle arrest. Although TIS functions as a solid tumor-suppressing barrier, the provocation of cell recurrence and therapy resistance by TIS suggests the need for exploration of combined senolytic (20) therapy with standard treatments for prostate cancer (3, 21).

**Table 1:** Therapeutic interventions inducing cellular senescence in prostate cancer and their underlying molecular pathways.

Therapy/stress type	Representative agents or conditions	Model system(s)	Dominant senescence pathways/mechanistic highlight	References
Chemotherapy (Microtubule & DNA-damaging)	Docetaxel, Doxorubicin, Mitoxantrone	LNCaP, DU145, PC3	NA damage response → activation of p53/p21 axis; increased ROS and p16^INK4A; irreversible G1 arrest	(22)
Radiotherapy (Ionizing Radiation)	Nutlin-3 + RT	22Rv1	Inhibits MDM2-p53 interaction → stabilization of p53 and amplification of radiation-induced senescence	(23)
Androgen Receptor (AR) Antagonists	Bicalutamide, Enzalutamide, Darolutamide	LNCaP, C4-2	AR signaling blockade → suppression, upregulation, and SASP activation	(24, 25)
Oncogene-Targeted Pathway Inhibitors	PI3K/AKT or MEK inhibitors	PC3, LNCaP	Restoration of PTEN/p27 signaling; induction of growth arrest via metabolic reprogramming	(26)
Epigenetic Modulators	Histone-deacetylase inhibitors (Vorinostat, Trichostatin A)	DU145, PC3	Histone hyperacetylation → reactivation of chromatin-linked senescence	(27, 28)
DNA-Repair Pathway Inhibitors	PARP inhibitors (Olaparib)	LNCaP, BRCA2-deficient PCa	Accumulation of DNA strand breaks → sustained p53/p21 response and senescence-like phenotype	(29, 30)
Oxidative/Metabolic Stress Inducers	Metformin, Resveratrol	LNCaP, PC3	Mitochondrial ROS production → AMPK activation and p53-mediated senescence stabilization	(31)

### 2.4 Epigenetic regulation of senescence in prostate cancer

There is also the possibility of epigenetic changes controlling the start and the maintenance of senescence in prostate cancer. The actions of DNMT1/3A/3B, the histone modifiers like EZH2, SIRT1, and HDAC1/2, and the chromatin remodelers all function in controlling the silencing and activation of transcription of certain genes related to senescence (32). During senescence, the formation of SAHFs (senescence-associated heterochromatin foci) suppresses the activation of the proliferation-promoting genes MCM3, PCNA, and Cyclin A. The absence of EZH2, or the inhibition of HDACs, reinforces the expression of p16 and p21, thus closing the gap and promoting the durable senescence arrest. EZH2 overexpression and SIRT1-mediated deacetylation, on the other hand, promote the bypass of senescence, which fuels tumor development and lineage plasticity. The latest research has shown that the activation of the AR signaling pathway interacts with chromatin modifiers to control the cessation of senescence, providing a pathway junction of hormonal signaling to epigenetic reprogramming (33).

## 3 Senescence as a tumor suppressor mechanism

Cellular senescence plays a pivotal role as a first line of defense in tumor suppression, meaning that it prevents cancerous cells from appearing by halting division in response to oncogenic or genotoxic stress. It was Hayflick and Moorhead who first described senescence as a replicative limit (34). Through the activation of canonical tumor suppressor pathways such as p53–p21 and p16–Rb, cells experiencing oncogenic stress, telomere shortening, oxidative damage, or DNA replication errors can undergo irreversible growth arrest. In the prostate, this senescence-associated growth arrest is particularly evident during early tumorigenesis and is often considered a failsafe against uncontrolled proliferation (3). One well-characterized mechanism involves oncogene-induced senescence (35), where activation of pathways such as RAS/MAPK triggers cellular stress responses that converge on p53 and p16INK4a to enforce a stable G1-phase arrest. In prostate epithelial cells, loss of PTEN—a common event in early lesions—can induce a senescence-like phenotype known as PTEN-loss-induced cellular senescence (PICS), acting as a brake on neoplastic transformation (3). These mechanisms contribute to the containment of early-stage lesions such as prostatic intraepithelial neoplasia (PIN). However, senescence is not merely a static endpoint. Recent evidence indicates that senescent cells, while growth-arrested, remain metabolically active and can influence their surroundings through senescence-associated signaling, most notably the SASP (36). The presence of persistent senescent cells in the prostate tumor microenvironment may paradoxically create conditions that support progression once immune clearance fails (3). This transition from a suppressive to a permissive role is influenced by the durability of cell cycle arrest, the composition of the SASP, and the responsiveness of the immune system. Therefore, while cellular senescence initially protects prostate tissue against tumorigenic insults, its long-term persistence may initiate new vulnerabilities (3).

## 4 Senescence-associated secretory phenotype

A defining hallmark of senescent cells is their ability to produce and release a complex set of secreted factors collectively known as the SASP. Unlike quiescent cells, senescent cells remain metabolically active and exert strong paracrine and autocrine effects on their environment through the SASP, which includes proinflammatory cytokines, chemokines, growth factors, matrix-remodeling enzymes, and extracellular vesicles. In the setting of prostate cancer (PCa), the SASP plays a paradoxical role; it initially supports tumor suppression through immune-mediated clearance of senescent cells, yet its chronic persistence contributes to tumor progression, tissue remodeling, and resistance to therapy (9).

The composition of the SASP is dynamic and context-dependent, varying according to the senescence trigger, cell type, and tissue microenvironment. Commonly secreted components include interleukins such as IL-1α, IL-6, and IL-8, as well as chemokines like CXCL1 and CCL2, matrix metalloproteinases (MMPs), and angiogenic factors such as vascular endothelial growth factor (VEGF). These factors are regulated by key intracellular signaling networks, most notably the DNA damage response (DDR), which activates transcriptional programs via nuclear factor-kappa B (NF-κB), CCAAT/enhancer-binding protein beta (C/EBPβ), and p38 MAP kinase. At the post-transcriptional level, the mammalian target of rapamycin complex 1 (mTORC1) plays an important role in controlling the translation of SASP components. Together, these pathways integrate genotoxic, oncogenic, and metabolic stress signals to maintain the senescent secretome (37).

In prostate cancer, SASP exerts profound effects on the tumor microenvironment. Initially, SASP components promote immune surveillance by attracting natural killer cells, macrophages, and cytotoxic T lymphocytes that are capable of clearing senescent or damaged cells. However, when senescent cells persist and SASP becomes chronically active, the secreted factors create a pro-tumorigenic niche. Interleukin-6 and interleukin-8, for example, activate the STAT3 signaling axis in neighboring epithelial cells, promoting proliferation, resistance to apoptosis, and epithelial-to-mesenchymal transition (EMT). Matrix metalloproteinases degrade the extracellular matrix, facilitating invasion and metastasis. Chemokines such as CXCL1 and CCL2 recruit immunosuppressive cells, including myeloid-derived suppressor cells (MDSCs) and M2-polarized macrophages, which contribute to immune evasion and sustained tumor growth (38).

Senescent stromal fibroblasts, often referred to as cancer-associated senescent fibroblasts (CASFs), are a major source of SASP in the prostate tumor microenvironment. These cells communicate with neighboring cancer cells through paracrine signaling loops, most notably involving IL-6/STAT3 and TGF-β/SMAD pathways. This intercellular communication enhances stemness features, therapeutic resistance, and lineage plasticity in tumor cells. Moreover, senescent cells release extracellular vesicles containing microRNAs such as miR-21 and miR-146, as well as metabolic enzymes that reprogram the metabolic profile of adjacent epithelial cells. This results in increased glycolytic activity, oxidative stress adaptation, and enhanced survival under therapeutic pressure (39).

The dual role of SASP in tumor biology depends largely on its temporal dynamics. Transient activation of SASP can be beneficial by promoting senescence surveillance and immune clearance, but prolonged SASP exposure leads to chronic inflammation, remodeling of the extracellular matrix, and escape from growth control mechanisms. This temporal shift from tumor-suppressive to tumor-promoting behavior underscores the core paradox of senescence in cancer. Whether SASP contributes to tumor suppression or progression depends on the immune competence of the host, the duration of the senescent state, and the molecular context of the tumor microenvironment (40).

Despite its central role in senescence biology, SASP remains difficult to characterize clinically. Its molecular profile is highly heterogeneous, and current biomarkers such as SA-β-gal, p16^INK4a, or serum IL-6 are insufficient to capture the dynamic nature and heterogeneity of SASP activity. Additionally, some SASP components may play beneficial roles in tissue repair or immune activation, complicating attempts to target SASP indiscriminately. The lack of robust, non-invasive tools to monitor SASP in real time presents a significant translational barrier. Approaches such as spatial transcriptomics, single-cell proteomics, and liquid biopsy-based SASP profiling are emerging as promising tools for addressing these challenges (41).

Given the critical role of SASP in sustaining tumor growth and therapy resistance in prostate cancer, there is growing interest in therapeutic interventions that target SASP-producing senescent cells. These strategies fall into two main categories: senolytics, which selectively eliminate senescent cells, and senomorphics (Senomorphics are agents that suppress or modulate the SASP without reversing the growth-arrested state of the senescent cell), which suppress or modulate the harmful components of SASP without affecting the senescent growth arrest (42).

## 5 Senescence and therapy resistance in prostate cancer

### 5.1 TIS after androgen deprivation therapy and radiotherapy

Androgen deprivation therapy (ADT) and radiotherapy (RT) are cornerstone treatments for localized and advanced prostate cancer (PCa). While both therapies aim to halt tumor proliferation, accumulating evidence shows that they may paradoxically induce TIS in residual cancer cells. This senescence is a stress response resulting from DNA damage, oxidative stress, or mitogenic imbalance caused by therapy. Unlike replicative or oncogene-induced senescence, TIS is often incomplete and heterogeneous, leading to a semi-permanent growth arrest that may later contribute to therapeutic resistance and disease recurrence (43).

**Figure 3:**
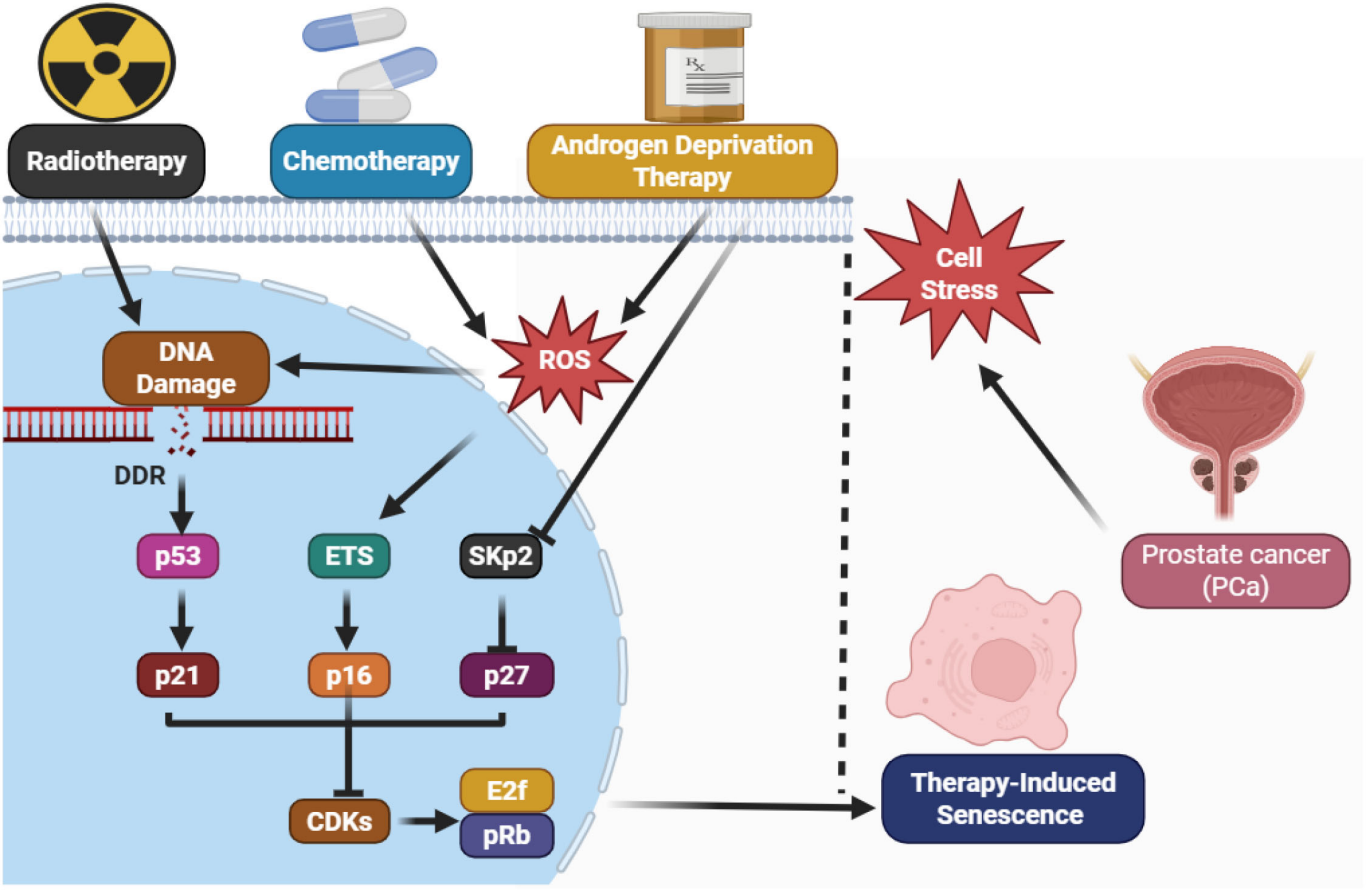
TIS after androgen deprivation therapy and radiotherapy.

During ADT, the suppression of androgen receptor (AR) signaling leads to growth arrest in AR-dependent prostate tumor cells. However, a subpopulation of cells—particularly luminal epithelial and stromal fibroblasts—undergoes partial senescence (44). These cells express classical senescence markers such as p16^INK4a, p21^CIP1/WAF1, and senescence-associated β-galactosidase (SA-β-gal), while also activating DNA damage response (DDR) pathways through ATM–p53–p21 signaling. Importantly, the senescence induced is often not terminal, creating a state of dormancy rather than clearance (**Figure 3**).

Radiotherapy, particularly when applied sequentially with ADT, induces more pronounced DNA double-strand breaks (DSBs), which result in persistent DDR activation. In p53-competent tumor cells, such as LNCaP and 22Rv1, sustained activation of p53 target genes enforces senescence through chromatin remodeling and cell cycle arrest. This senescence is also associated with the induction of the SASP, a pro-inflammatory state that alters the tumor microenvironment. In contrast, p53-deficient or mutant cells (e.g., DU145, PC3) exhibit radioresistance, reduced TIS, and are more likely to evade growth arrest altogether (45).

One of the most critical mechanisms through which TIS promotes therapy resistance is via SASP-mediated JAK/STAT3 signaling. In particular, IL-6 and IL-8, secreted by senescent tumor or stromal cells, activate STAT3, which contributes to the reactivation of AR and its co-regulators, even in an androgen-deprived environment. This AR reactivation enables residual tumor cells to proliferate despite therapy, marking a key pathway to CRPC (46).

Thus, although TIS initially impedes tumor growth, the persistence of senescent cells and their secretory phenotype facilitates the emergence of therapy-resistant clones. These cells contribute to a pro-tumorigenic relapse niche by promoting survival signaling, immune evasion, and stemness acquisition (47).

### 5.2 Persistent SASP and tumor relapse

While TIS initially halts tumor growth, the persistence of senescent cells, particularly those that adopt a SASP, plays a central role in promoting tumor relapse. The SASP is composed of a wide array of cytokines (e.g., IL-6, IL-8), chemokines (e.g., CXCL1, CCL2), growth factors, and matrix-remodeling enzymes, which together reshape the tumor microenvironment in ways that favor cancer progression.

Mechanistically, the chronic activation of SASP is maintained through pathways such as NF-κB, C/EBPβ, mTOR, and sustained DNA damage response signaling. These factors create a pro-inflammatory niche, supporting the survival and proliferation of both senescent and non-senescent tumor cells. In prostate cancer, IL-6/STAT3 signaling is critical; it fosters resistance to androgen deprivation by reactivating AR-related transcriptional programs and enhancing tumor cell plasticity (48). The SASP also acts in a paracrine manner, influencing nearby stromal and immune cells. For instance, SASP-mediated secretion of CXCL1 and CCL2 recruits myeloid-derived suppressor cells (MDSCs) and M2-polarized macrophages, both of which contribute to immunosuppression, allowing senescent or dormant tumor cells to evade immune surveillance. This immunosuppressive microenvironment reduces the likelihood of senescent cell clearance, allowing these cells to persist and continue modulating the tissue landscape (49).

Furthermore, persistent SASP signaling contributes to epithelial-to-mesenchymal transition (EMT) and extracellular matrix (ECM) remodeling, which enhances tumor cell motility, invasion, and resistance to apoptosis. These changes not only drive local tumor regrowth but also increase the potential for metastasis, particularly in advanced stages of prostate cancer. Therefore, while the senescence program was initially triggered as a protective response, the failure to clear senescent cells and the sustained activity of SASP components collectively create a pro-tumorigenic feedback loop. This loop supports residual disease, promotes immune escape, and facilitates aggressive relapse, underscoring the urgent need to target persistent SASP in prostate cancer management therapeutically (50).

### 5.3 Stemness reprogramming post-senescence

Prostate tumor cells may be able to momentarily reverse the senescence state due to chronic SASP signaling or environmental stress. NOTCH1 pathways, leading to the reprogramming of neighboring epithelial cells to cancer stem cells (CSCs) that express the stemness markers CD44, SOX2, and NANOG (51). Chronic exposure to SASP cytokines, particularly IL-6 and IL-8, enhances STAT3-mediated transcription of stemness-associated genes such as SOX2 and NANOG. This contributes to the development of CSC-like phenotypes, enabling tumor recurrence and multi-drug resistance. Acquisition of stemness by previously senescent cells enables tumor regrowth and increased regenerative potential while also fostering multi-drug resistance. In addition, these cells undergo a metabolic shift that includes a predominance of glycolysis and adaptation to oxidative stress, both defining characteristics of androgen-independent or castration-resistant prostate cancer (CRPC). Therefore, TIS may momentarily stem tumor expansion, but the TIS state creates a quiescent, therapy-resistant niche wherein the tumor eventually relapses, and the cancer spreads (52).

### 5.4 Conceptual integration and therapeutic implications

The paradoxical role of cellular senescence in prostate cancer underscores the need for therapeutic strategies that can selectively enhance its tumor-suppressive functions while limiting its tumor-promoting effects. Senescence can be induced by radiation, chemotherapy, or androgen deprivation therapy, yet unresolved or persistent senescent cells often contribute to tumor relapse through SASP-driven inflammation, immune modulation, and cellular reprogramming (53). Given these dynamics, therapeutic approaches that modulate or eliminate senescent cells have become an area of active research. Combined treatment strategies—those that induce senescence to arrest tumor growth while concurrently targeting residual senescent cells or their secretory phenotype—may represent a promising avenue to minimize therapy-induced resistance and recurrence (54).

## 6 Targeting senescence: therapeutic opportunities and risks

**Figure 4:**
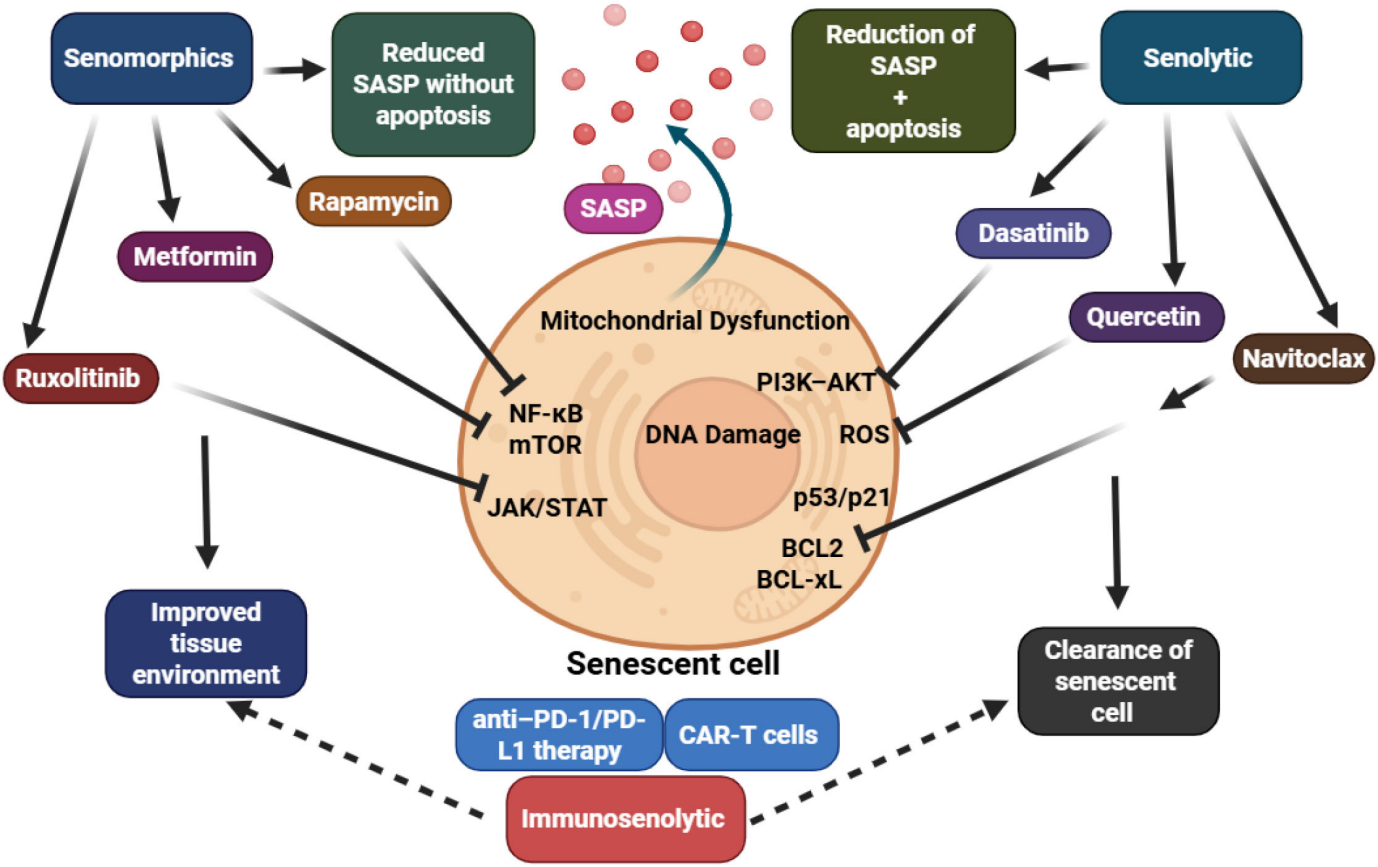
Senolytics eliminate senescent cells via BCL-2/BCL-xL inhibition, while senomorphics suppress SASP through mTOR, JAK/STAT, and NF-κB pathways. Both strategies aim to counter SASP-driven resistance and relapse, though their efficacy and safety remain context dependent.

The evolving understanding of senescence in prostate cancer has unveiled new therapeutic opportunities aimed at targeting senescent cells or modulating their harmful secretory output. Senescence can act as both a suppressor and promoter of prostate cancer depending on context, timing, and cellular compartment (8). This dual role presents a significant therapeutic paradox: while senescence induction through therapies like ADT and radiotherapy RT may halt proliferation, the long-term persistence of senescent cells and their secretory phenotype—known as the SASP—can foster immune evasion, resistance, and relapse. Therefore, an emerging therapeutic approach involves selectively removing or neutralizing senescent cells through the use of senolytic or senomorphic agents (**Figure 4**) (8).

Senolytic agents are designed to selectively induce apoptosis in senescent cells by disrupting pro-survival pathways. Among the most studied senolytics in prostate cancer research are navitoclax (ABT-263), dasatinib, and quercetin (55). These agents target proteins such as BCL-2 and BCL-xL that are upregulated in senescent cells, allowing for their removal. In preclinical prostate cancer models, navitoclax has shown promise in clearing senescent cells induced by ADT or RT, thereby reducing SASP load and improving therapeutic outcomes. However, navitoclax’s progression to clinical use has been hindered by its on-target toxicity, particularly the induction of thrombocytopenia, which underscores the need for improved delivery mechanisms or combination strategies to enhance its selectivity (55).

In contrast, senomorphic drugs do not eliminate senescent cells but rather aim to suppress or reprogram the SASP. Agents such as rapamycin, metformin, and JAK/STAT pathway inhibitors have been shown to reduce the pro-inflammatory and tumor-promoting features of senescent cells while preserving their growth-arrested state. Rapamycin, an mTOR inhibitor, has demonstrated efficacy in dampening SASP expression and preventing AR reactivation, particularly in the context of therapy-induced senescence. Similarly, JAK inhibitors like ruxolitinib can disrupt IL-6 and IL-8-driven SASP signaling, which has implications for blocking AR reactivation and immunosuppressive cell recruitment (56).

Combination strategies are gaining traction as a rational way to address the complexity of senescence. For instance, combining senolytics with ADT or RT may prevent the long-term accumulation of senescent cells after therapy, while adding senomorphics could mitigate the harmful systemic effects of SASP without compromising the beneficial growth arrest (57). These synergistic approaches offer a more tailored solution, particularly in managing high-risk or CRPC. However, the success of such interventions depends heavily on optimizing the timing of administration, identifying biomarkers that indicate the presence and type of senescent cells, and balancing the risks associated with removing cells that may serve protective roles in tissue remodeling or immune surveillance.

Despite promising preclinical data, clinical translation remains limited. Few senescence-targeting agents have progressed beyond early-phase trials, and data specific to prostate cancer are particularly sparse. The field continues to face challenges in patient stratification, mainly due to the absence of reliable, non-invasive senescence biomarkers. Although markers such as p16^INK4a, SA-β-gal, and certain SASP components are used experimentally, they lack the specificity, consistency, and clinical validation needed for therapeutic decision-making (58). Furthermore, distinguishing tumor cell senescence from stromal or immune cell senescence *in vivo* remains a critical obstacle to therapeutic precision.

In future clinical applications, successful integration of senescence-targeted therapy into prostate cancer treatment will likely depend on the development of robust senescence profiling tools, tissue-specific delivery systems, and rational combination strategies involving immunotherapy, chemotherapy, and hormonal agents. While the senescence program presents a therapeutic vulnerability, it also poses significant risks if not precisely controlled. Therefore, the path forward requires both scientific innovation and careful clinical stewardship to fully harness the benefits of senescence manipulation while minimizing its long-term liabilities.

## 7 The paradoxical balance — integrative perspective

**Figure 5:**
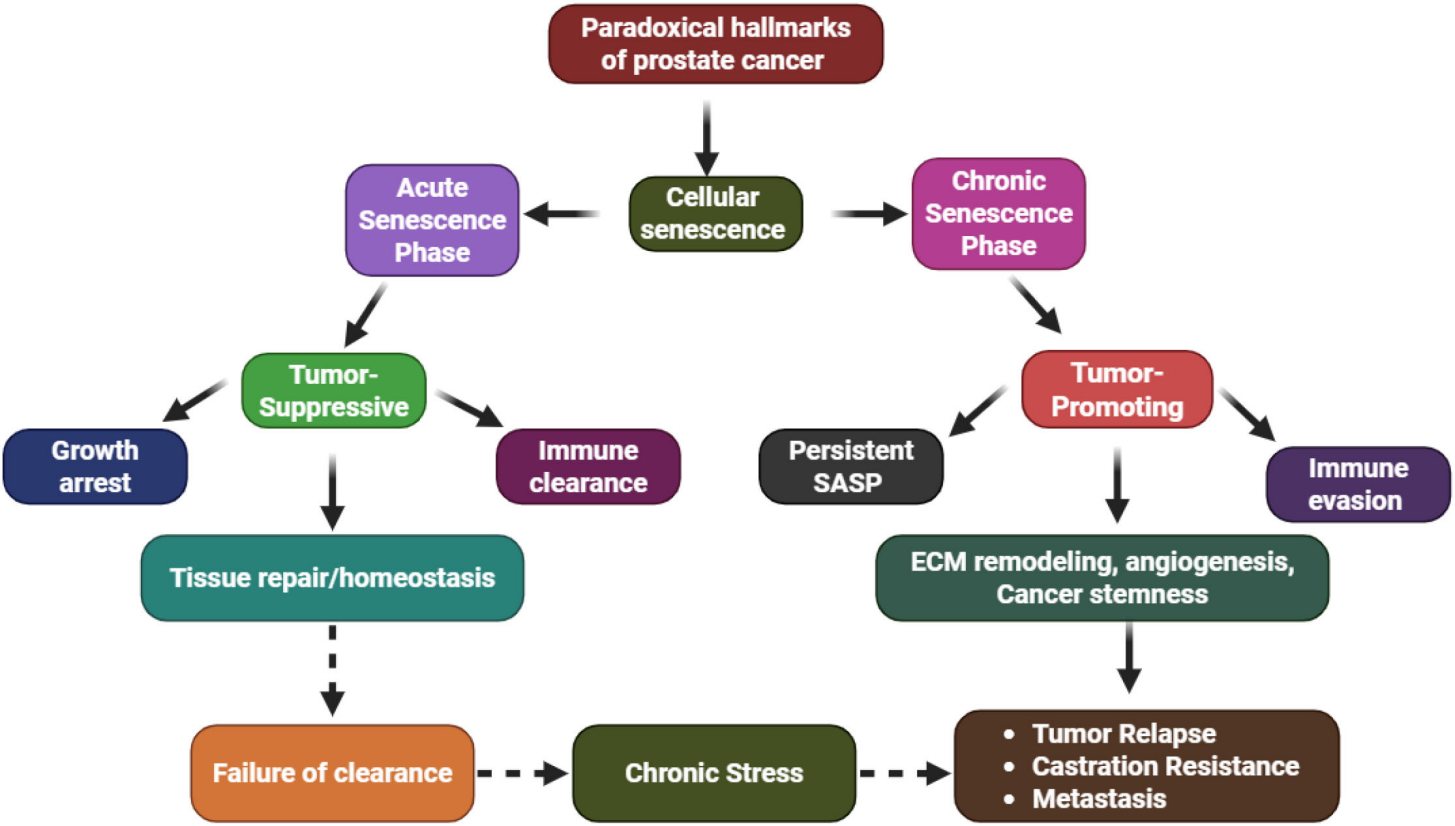
The paradoxical balance of senescence in prostate cancer.

Cellular senescence is one of the most paradoxical hallmarks of prostate cancer biology. The phenomenon could limit malignant progression or quietly aid it, depending on the moment in time and the surrounding milieu (**Figure 5**). During the initial stages of tumorigenesis, senescence acts as a powerful barrier to oncogenic transformation, stopping the assault on proliferative control by damaged or pre-malignant cells via the p53–p21 and p16–Rb pathways (36). This growth arrest ensures the preservation of genomic stability and enables immune-mediated clearance of senescent cells by cytotoxic T lymphocytes, macrophages, and NK cells. However, as tumors evolve, the very same senescence programs may become maladaptive; uninvolved or incomplete senescence may transform from a mere cytostatic checkpoint into a pro-tumorigenic hypoxic niche that supports relentless chronic inflammation, tissue remodeling, and immune evasion (59).

The effect of prostate cancer senescence as biogenic, promotive, or neutral depends on three broad factors: the microenvironment, the composition of the immune system, and the permanence of the senescent condition. In a situation where the senescent microenvironment expands as a result of time, prolonged protective senescences will take effect as tissue maintenance will occur without the tissue becoming overly invasive and destructive (60). In contrast, senescent cells can be formed as a result of external stressors (androgen deprivation, radiation, oxidative and cellular senescence) and will break through the functional and secured senescent domain (capture and contain) and begin to secrete IL-6, IL-8, TGF-β, and VEGF. The IL-6-dominant secretome alters the cancer microenvironment through the recruitment of MDSCs and M2 macrophages, enabling cocooning and shielding the tumor from immune-mediated lysis. In prostate cancer, the IL-6-stimulated SASP silencing of certain genes within the IL-6/STAT3 pathway activates castration-resistant lineage plasticity, driving the disease, which exemplifies how a protective SASP process shifted derivative therapy resistance in prostate cancer (61).

From the perspective of the immune system, senescence may serve different functions, positive or negative, depending on the context. In early phases of senescence, the immune mechanisms aimed at eliminating senescent cells are activated, but chronic or TIS seems to lead, at least in the end, to immune exhaustion and tolerance (62). The combination of senescent epithelial cells, cancer-associated fibroblasts, and immune infiltrates ultimately determines whether senescence will continue to function as a tumor-suppressive barrier or provide a fertile ground for tumor growth. The phenomenon of immune cells communicating with senescent cells embodies what has been termed ‘senescence plasticity (the dynamic switching between tumor-suppressive and tumor-promoting roles based on cellular and environmental context),’ the same biological mechanisms leading to disparate functions, determined by the timing and context of the surrounding environment (63).

To visualize the progression of prostate cancer through the lens of senescence concepts, one can think of the progression consisting of different states of senescence, starting with the protective oncogene-induced senescence, moving to persistent therapy-induced senescence, and, ultimately, reaching SASP, which promotes the tumor and relapses. In the described progression, the pivot point is the persistent senescence, which transforms the state from suppression to promotion. Immune system-cleared senescence supports tumor control, while unresolved or chronic forms of senescence promote neoplastic regrowth, dedifferentiation, and metastasis, fostering the microenvironment (64). This leads to the integrative model of senescence plasticity in prostate cancer. Acute Senescence (Tumor-Suppressive Phase): p53/p21 and 16/Rb driven arrest, immune surveillance, and tissue homeostasis are characterized by chronic senescence (tumor-promoting phase), which is driven by sustained SASP, stromal activation, immune suppression, and cancer stemness reprogramming. Microenvironmental stress, therapy-induced DNA damage, and the failure to clear senescent cells drive the plastic transition between states. The duality of senescence stands in the contextual and temporal relations. Senescence, designed to protect, can also betray the host if the tumor suppressive state is not cleared or if it is chronic. No matter what, the future therapeutic strategies on senescence in cancer cells must leave room for control and timeliness in order to maintain the dormant state. Prostate cancer provides an example to highlight the importance of controlling the state of senescence to prevent chronic tumor-promoting progression (65).

## 8 Future directions

Prostate cancer research has made considerable progress in the comprehension of the biological fundamentals of cellular senescence, but there still remain considerable obstacles in defining an exact temporal order of when senescence is a benign, tumor-suppressing phenomenon, and when it becomes a malignant, tumor-promoting phenomenon. In particular, distinguishing transient, reparative senescence from persistent, maladaptive senescence remains to be solved. Similarly, the reliance on static senescence biomarkers—such as SA-β-gal, p16^INK4A, and p21^CIP1—has been problematic (66). While static biomarkers have their merit, they ultimately fail to reflect the spatial and temporal evolution of senescent cell populations. Thus, future research should address the characterization of senescent subpopulations within the functional and spatial heterogeneity *in vivo*, through an integration of multi-omic single-cell profiling with lineage tracing and spatial transcriptomics.

Identifying reliable and flexible biomarkers that can assess cellular senescence in patients over time is an urgent necessity. Currently used biomarkers, such as SA-β-gal, p16^INK4a, and p21^CIP1, are largely static and context-dependent. They fail to reflect the dynamic evolution or clearance of senescent cell populations and cannot distinguish between protective and pathological senescence. Additionally, variability in expression across cell types and tumor grades undermines their reliability as predictors of therapeutic response. Liquid biopsy approaches, metabolic signatures, and spatial transcriptomic profiling are promising alternatives but remain largely experimental. Certain circulating SASP constituents—IL-6, CXCL8, and MMPs— exosome miRNAs, and certain metabolic profiles, appear promising as potential minimally invasive biomarkers for detecting TIS and for the early relapse of the disease. These markers could become part of liquid biopsies for detecting senescence persistence, allowing real-time adaptive treatment modifications. Additionally, the use of molecular imaging, including senescence-activated β-galactosidase probes and p16 or SAHF-structure PET tracers, provides novel ways to assess senescent cell burdens in all metastatic locations (65).

The next therapeutic approach seeks to target senescence more specifically. The new class of senolytic and senomorphic drugs seeks to eliminate harmful senescent cells or neutralize the harmful secretions of senescent cells, while leaving the benefits of senescence intact. Blockade of the BCL-2 family by Navitoclax, along with other HSP90 inhibitors, and FOXO4–p53 disruptors, which specifically target stromal cells, is undergoing preclinical testing with BCL-2 inhibitors and is claimed to eliminate stromal cancer cells. Proposed therapeutic agents that act as senomorphic to the SASP are JAK/STAT inhibitors or NF-κB pathway modulators that are currently being tested for the SASP-induced inflammatory and immunosuppressive effects. The primary issue is how to fine-tune the selectivity and the therapeutic window to avoid the relapse of tumors in the cascade of events that have to occur after senescence is therapeutically induced.

The incorporation of senescence biology into the frameworks of precision oncology remains an unprecedented stride in the discipline. For the treatment of prostate cancer, there exists the possibility of a “senescence signature–guided” approach to therapeutic stratification, wherein the senescent cell populations inform the use of senolytic or immunomodulatory therapy. The combination of senescence-inducing therapies, like radiotherapy and androgen receptor blockade, with therapies that clear senescence, like immune checkpoint therapies or senolytics, could transform treatment paradigms for patients with advanced disease. The goal is a system-level vision for oncology in which senescence, rather than merely a byproduct of a therapy, is an active, measurable, and targeted element in the prostate cancer evolutionary framework.

The management of senescence’s spatiotemporal attributes and control of senescence’s duality will dictate whether the aged program of a cell will become a therapeutic paradox or a cornerstone of curative oncology. Actionable precision medicine will necessitate the integration of biomarker-driven clinical trials, AI-assisted modeling, and patient-specific senescence profiling to move the discipline from descriptive biology.

## 9 Conclusion

Cellular senescence in prostate cancer poses a logical dilemma: a program aimed at containing malignant transformation paradoxically promotes malignant transformation under chronic or dysregulated conditions. The opposing effects of the senescence phenomenon suggest remarkable situational signaling. During the early stages, senescence is crucial in preventing malignancy because, in addition to imposing cell cycle arrest through the p53/p21 and p16/RB pathways, it also triggers an immune response to clear out senescent, damaged cells. Conversely, in more advanced stages, the chronic and persistent senescent cells and, particularly, the SASP, drive chronic inflammation, epithelial-mesenchymal transition, and escape from therapy. To leverage the paradox of senescence in malignancy, context will be critical: active, molecular mechanisms, duration of persistence, and immune presence or obstruction. The future management of prostate cancer will depend on the ability to assess the transient, non-proliferative, yet metabolically active state of the tumor driven by unresolved senescence. The practical and biological application of systems theory to senescent cells will revolutionize the control of tumor evolution in prostate cancer p53.
